# Complete genome sequence of a co-cultured Phycisphaerae bacterium of the phylum Planctomycetota, strain 2_2a_38c

**DOI:** 10.1128/mra.00653-26

**Published:** 2026-10-21

**Authors:** Zhiwei Peng, Yumi Shimomura, Kazuki Mori, Setsu Kato, Yutaka Nakashimada, Kosuke Tashiro, Yoshiteru Aoi

**Affiliations:** 1 Graduate School of Integrated Sciences for Life, Hiroshima University 12801, Hiroshima, Japan; 2 Graduate School of Systems Life Sciences, Department of Bioscience and Biotechnology, Kyushu University 12923, Fukuoka, Japan; 3 Computational Bio-Big Data Open Innovation Laboratory, National Institute of Advanced Industrial Science and Technology, Tokyo, Japan; 4 Seto Inland Sea Carbon-neutral Research Center, Hiroshima University 12801, Hiroshima, Japan; Montana State University, Bozeman, Montana, USA

**Keywords:** Phycisphaerae, Planctomycetota

## Abstract

We report the 4.1-Mb complete genome sequence of a novel member from the class Phycisphaerae (Phycisphaerae bacterium strain 2_2a_38c) of the phylum Planctomycetota, which was sequenced from a co-culture with *Nocardioides* sp. strain 2_2a_38e, originated from the forest soil in Hiroshima, Japan.

## ANNOUNCEMENT

Bacteria from the class Phycisphaerae are widely distributed across diverse ecosystems, but most of them remain uncultivated (1). We obtained a co-culture consisting of Phycisphaerae bacterium strain 2_2a_38c and *Nocardioides* sp. strain 2_2a_38e (PX472977.1) from a forest soil sample (0–10 cm depth; N34°24′03.03″, E132°42′44.67″). Approximately 30 g of soil was collected in a sealable plastic bag, transported to the laboratory at room temperature, and processed within 30 min. Microbes were extracted and purified as described previously (2), except that 1× phosphate-buffered saline was used for cell extraction. The extracted cells were encapsulated and cultivated using gel microdroplet (GMD) aggregation cultivation system (2). GMDs physically separate cells while permitting chemical interactions between microorganisms in neighboring GMDs.

After cultivation, GMDs were stained with SYBR Green I, fluorescence-positive GMDs were isolated by fluorescence-activated sorting using a Cell Sorter (Sony, Japan). Two GMDs were deposited into each liquid culture containing 10% R2A broth. During subsequent incubation, microbial cells proliferating within the GMDs grew out into the surrounding liquid medium and continued to grow, ultimately allowing the establishment of a stable two-member co-culture. The co-culture was subsequently scaled up in liquid medium to a final culture volume of 5 mL, yielding sufficient biomass for further analysis.

Genomic DNA was extracted from the entire cell pellet (approximately 10^9^ cells), using the NucleoSpin Microbial DNA kit (Takara Bio Inc., Japan) according to the manufacturer’s instructions. Sequencing was performed using the Illumina MiSeq (2 × 300 bp) and Nanopore MinION Mk1C platforms. For Illumina paired-end sequencing, DNA was sonicated to an average of 600 bp without additional size selection. The libraries were prepared using the NEBNext Ultra II DNA Library Prep Kit (New England Biolabs, USA). A total of 4,739,756 reads were obtained. Reads were processed with fastp v0.23.4 (3) using -q 30, -l 200, -n 0,-3, --detect_adapter_for_pe, and -w 8. For Nanopore sequencing, libraries were constructed using SQK-LSK109 Ligation Sequencing Kit (ONT, UK) and sequenced on R9.4.1 (FLO-MIN106) flow cell, producing 100,045 raw reads (N_50_, 12,327 bp). Guppy v3.4 fast-model base calling (4) enabled real-time processing on the Mk1C, and only pass reads (Q score > 7) were retained. Adapters were trimmed using Porechop v0.2.4 (5). Illumina and Nanopore reads were assembled and circularized using Unicycler v0.5.1 (6), and the resulting assemblies were polished with the Illumina reads using Pilon v1.24 (7), rotated to begin at *dnaA*, and annotated using DFAST v1.3.6 (8). Default parameters were used unless otherwise specified.

**Fig 1:**
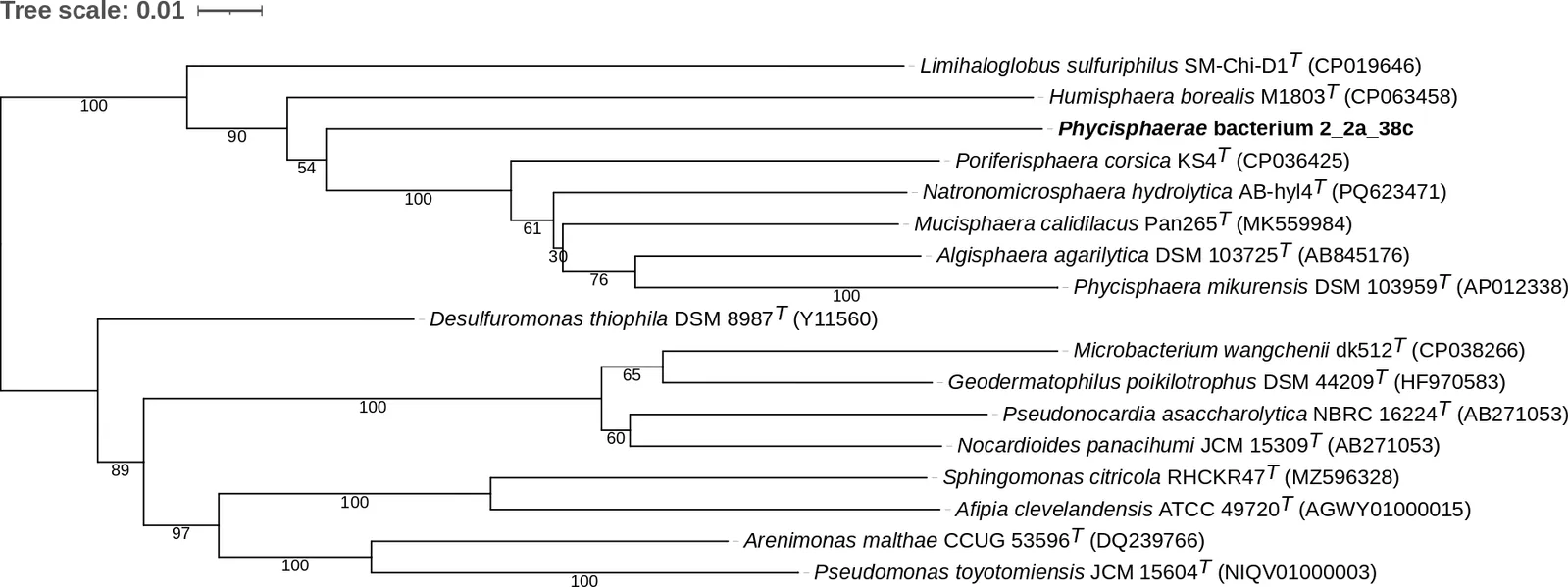
Phylogenetic tree inferred with FastME 2.1.6.1 (11) from GBDP distances calculated from 16S rRNA gene sequences using TYGS. Branch lengths are scaled according to GBDP distance formula d5. Numbers at the branching points indicate GBDP pseudo-bootstrap support values based on 100 replicates. The tree was midpoint-rooted. *T*, type strain.

The assembly corresponding to Phycisphaerae bacterium strain 2_2a_38c comprised a 4,125,036-bp circular chromosome (61.2% G + C content; 100× coverage) and was 100% complete according to CheckM v1.2.2 (9). DFAST predicted 3,468 genes, including 3,417 coding sequences, 3 ribosomal RNAs (rRNAs), and 48 transfer RNAs. TYGS analysis (10) of the chromosome-derived 16S rRNA gene identified *Poriferisphaera corsica* KS4 as the closest 16S match (87.38%); the 16S GBDP tree is shown in Fig. 1. A separate whole-genome TYGS analysis identified *Limihaloglobus sulfuriphilus* SM-Chi-D1 as the type strain with the smallest GBDP distance; the corresponding dDDH estimate was 22.7% using formula d4.

## Data Availability

The complete genome sequence of Phycisphaerae bacterium strain 2_2a_38c has been deposited in GenBank under accession numbers AP046646. The version of the genome described in this paper is the first version. Raw sequence data from Illumina MiSeq and MinION are available at DDBJ Sequence Read Archive under DRA accession numbers DRR1046605 and DRR1046606, respectively.
